# Efficacy of micro–nano bubble enhanced immobilized *Chlorella vulgaris* in the removal of typical antibiotics†

**DOI:** 10.1039/d5ra02082d

**Published:** 2025-06-16

**Authors:** Tao Zhu, Mengyao Jing, Jianping Zhang, Hui Li, Min Zhou, Guijuan Li

**Affiliations:** a Henan College of Transportation Zhengzhou 450008 Henan China; b School of Water and Environment, Chang'an University Xi'an 710054 China; c Key Laboratory of Subsurface Hydrology and Ecology in Arid Areas, Ministry of Education, Chang'an University Xi'an 710054 China; d Key Laboratory of Eco-hydrology and Water Security in Arid and Semi-arid Regions of Ministry of Water Resources, Chang'an University Xi'an 710054 China; e Henan Transport Investment Group Co., Ltd Zhengzhou China; f Ocean University of China Qingdao 266100 Shandong China zhoumin2023@126.com; g Henan Provincial Department of Transport Zhengzhou 45000 Henan China

## Abstract

Antibiotic pollution poses a global environmental challenge, with effective removal technologies for different antibiotic types still lacking. This study investigates an innovative micro–nano bubble (MNB)-augmented immobilized *Chlorella vulgaris* system for remediating groundwater contaminated with sulfadiazine (SD) and chloramphenicol (CAP) antibiotics. Key parameters, including initial concentration (5–30 mg L^−1^), algal bead density (0.25–4 beads per mL), aeration time (5–30 min), and coexisting ions, were evaluated. SEM and FT-IR analyses revealed removal mechanisms. Results showed MNBs significantly improved microalgal biomass and removal efficiency (SD: 79.97%; CAP: 93.92%). SD elimination primarily depends on initial concentration and aeration, while CAP removal shows stronger ionic environment dependence. FT-IR confirmed stronger interactions (electrostatic attraction, surface adsorption) between algae and CAP. The technology showed particular effectiveness for CAP, achieving over 90% removal through MNB-algae synergy, providing valuable insights for targeted antibiotic remediation strategies.

## Abstract 2

Antibiotic pollution poses a global environmental challenge, with effective removal technologies for different antibiotic types still lacking.

## Graphical abstract

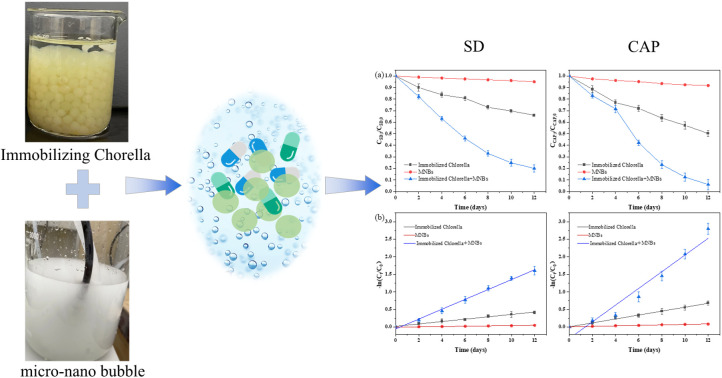

## 1 Introduction

The increasing prevalence of antibiotic contamination and associated resistance genes has become a critical environmental issue.^1–4^ Multiple factors contribute to this problem, including partial metabolic breakdown in organisms^5,6^ and inadequate removal by conventional wastewater treatment.^7^ These contaminants migrate to groundwater through various pathways, ^8,9^ threatening aquatic ecosystems and potable water resources.^10,11^ Current remediation approaches face substantial challenges: ^12,13^ biological methods risk inducing resistance, ^14^ chemical treatments often prove costly and generate hazardous byproducts, ^2^ while physical techniques suffer from interference and high operational expenses.^15^ This situation demands innovative solutions for groundwater remediation that are simultaneously effective, economical, and environmentally sustainable.

Microalgae technology has the potential to remove pollutants and nutrients, reduce biochemical oxygen demand, efficiently capture CO_2_, be eco-friendly, and produce high-value products, so it has become a promising alternative.^16,17^ Frascaroli *et al.* (2024)^18^ used three microalgae (*Auxenochlorella protothecoides*, *Tetradesmus obliquus*, and *Chlamydomonas acidophila*) to effectively remove the mixture of seven antibiotics (ciprofloxacin, clarithromycin, erythromycin, metronidazole, ofloxacin, sulfamethoxazole and trimethoprim). At the same time, since microalgae are non-target organisms of antibiotics,^19^ microalgae show significant adaptability during antibiotic treatment.^20^ In particular, heterotrophic microalgae have higher biomass production, extracellular polymeric substances (EPS) accumulation, and pollutant removal efficiency than photosynthetic autotrophic microalgae.^21^ Similar situations exist in other microorganisms, such as heterotrophic denitrifying bacteria.^22^ In this sense, microalgae can fully adapt to the dark environment of groundwater.

However, despite the excellent performance of microalgae in antibiotic removal, its removal efficiency is still affected by many factors, such as microalgae species, antibiotic species and concentration, dissolved oxygen (DO) concentration, light intensity, *etc.*^23^ Therefore, in practical applications, the selection of appropriate microalgae species and optimization of process conditions are key to improving the removal efficiency of antibiotics. To further increase the efficiency of antibiotic removal by microalgae, researchers have begun to explore new technological tools in recent years.^24^ For instance, micro–nanobubble (MNB) technology has emerged as a prospective support, with potential to enhance performance across various bioprocesses.^25,26^ Miao *et al.* (2024)^27^ demonstrated that the application of MNBs by using novel oxygenated MNBs loaded with micropore biochar to stimulate indigenous aerobic denitrifying bacteria could effectively alleviate hypoxia, enhance the overall collaboration among microorganisms, and promote the expression of relevant genes. Temesgen *et al.* (2023)^28^ applied nanobubbles to the aeration mechanism of a semi-intermittent bioreactor and improved the biodegradation rate of organic matter in wastewater from 5.83 to 17.5 mg L^−1^ h^−1^. Compared to conventional bubbles, MNBs exhibit faster mass transfer rates and lower rise velocities due to their small diameter and high internal pressure.^29^ Introducing MNBs into microalgae culture systems significantly enhances the efficiency of oxygen transfer from internal bubbles to the external water column, generating higher DO concentrations to improve the low-oxygen environment of groundwater and promoting the growth and metabolic activities of microalgae, which can accelerate their degradation of antibiotics.

In addition, the application of immobilization technology also provides innovative approaches for antibiotic removal by microalgae. Immobilizing microalgae onto carriers improves their stability and reusability in wastewater treatment.^30,31^ Xie *et al.* (2020)^17^ showed that immobilized *Chlorella* outperformed the free state of *Chlorella* in antibiotic removal.

Sulfonamides and amphenicols account for 12% and 8% of global antibiotic usage, respectively, and their environmental occurrence concentrations and associated ecological risks exhibit significant differences.^8^ This study selected sulfadiazine (SD, representing sulfonamides) and chloramphenicol (CAP, representing amphenicols) as representative antibiotics. The aqueous residual concentration of SD can reach the μg L^−1^ level, while CAP is more prone to accumulation in sediments.^32^ The strong lipophilicity of CAP facilitates its adsorption onto algal cell membranes, whereas the high water solubility of SD enables its diffusion primarily through the aqueous phase. The two antibiotics exhibit significant differences in chemical structure, physicochemical properties, and degradation pathways, making them effective indicators for assessing the system's removal characteristics for different types of antibiotics. In this study, the widely used green algae genus *Chlorella vulgaris* (*C. vulgaris*) was employed as the experimental material to investigate the effect of MNBs on the removal of SD and CAP by immobilized *C. vulgaris*.^33,34^ We examined the effects of various factors on removing antibiotics from groundwater using MNBs-enhanced immobilized *C. vulgaris* and determined the dominant factors through correlation analysis. Afterward, two characterization methods, scanning electron microscopy (SEM) and Fourier transform infrared spectroscopy (FT-IR), were used to analyze the antibiotic removal pathway and determine the ESP content to reveal the antibiotic removal mechanism further. This work provides new insights for applying immobilized microalgae technology in groundwater pollution remediation, contributing to upgrading traditional biotechnology.

## 2 Materials and methods

### 2.1 Preparation of micro–nano bubbles

The micro–nano bubble generator (ZJC-NM-200L, Shanghai Zhongjing Environmental Technology Co., Ltd) utilizes multiphase vortex gas–liquid shear and swirl countercurrent technology (Fig. SI1†). During the experiment, the operating pressure of the device was maintained at 0.3–0.35 MPa, with an air intake of approximately 100 mL min^−1^. The dissolution rate of the generated MNBs exceeded 95%. The resulting bubbles exhibited a size distribution of 100–1000 nm in diameter and demonstrated remarkable stability, persisting for over one week in an aqueous solution (Fig. SI2 and SI3†).

### 2.2 Materials

The *Chlorella vulgaris* strain used in the experiment, with the strain number FACHB-8, was purchased from the Freshwater Algae Culture Collection at the Institute of Hydrobiology, Chinese Academy of Sciences, Wuhan. Antibiotics were chromatographically pure and purchased from Shanghai Yuanye Biotechnology Co., Ltd, China, and their physicochemical properties are shown in Table 1. The simulated groundwater samples were prepared in the proportion of 0.1665 g per L CaCl_2_, 0.1952 g per L MgSO_4_, 0.3884 g per L NaHCO_3_, and 0.0160 g per L KNO_3_, referring to the literature.^35^

**Table 1:** Physicochemical properties of antibiotics used in the study

Name	Chemical formula	Molecular	Molecular weight (g mol^−1^)	log *K*_ow_^a^	p*K*_a_^a^
Sulfadiazine (SD)	C_10_H_10_N_4_O_2_S	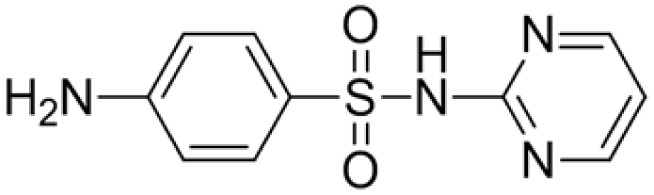	250.277	−0.09	6.36
Chloramphenicol (CAP)	C_11_H_12_Cl_2_N_2_O_5_	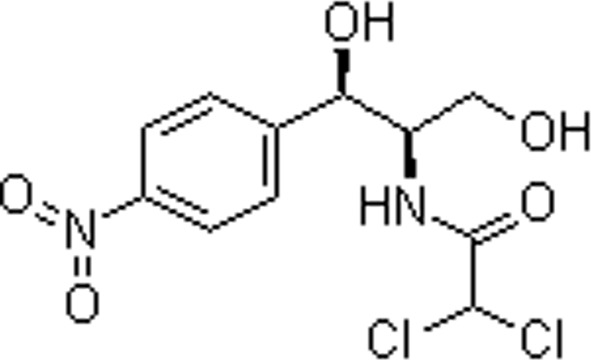	323.129	1.14	11.03

a Experimentally determined values.

### 2.3 Immobilization and de-immobilization of *C. vulgaris*

*C. vulgaris* was inoculated into BG-11 liquid medium (containing NaNO_3_ 1.5 g L^−1^; other components are listed in Table SI1†) and maintained at pH7–7.5. The cultures were continuously subculture for 2–3 cycles in an illuminated incubator (25 °C, 3000–4000 lux, light–dark cycle of 12 h : 12 h). OD_680_ was monitored daily, and the standard for activity recovery was defined as a stable biomass growth rate (ΔOD_680_ per day > 0.15).

The algae cells were collected by centrifugation and resuspended in sterile deionized water, and the absorbance of the algae solution was adjusted to 0.3. The algae solution was mixed with 5% sodium alginate solution at a volume ratio of 1 : 1. The mixed droplets were added to 2% CaCl_2_ (w/v) solution at a rate of 3.6 mL min^−1^ using a peristaltic pump to form immobilized algal beads with a diameter of about 3–4 mm. The beads were hardened for 4 h, separated using filter paper, and rinsed with sterile water for subsequent use. Each algal bead contained approximately 0.0524 mL of algal solution and weighed about 0.05 g. For de-immobilization, the algal beads were placed in a 10% (w/v) sodium citrate solution and gently shaken in an oscillator until the beads were dissolved entirely.^17^

### 2.4 Experimental design

#### 2.4.1 Antibiotics removal experiment

Three replicates of two antibiotic solutions with an initial concentration of 10 mg L^−1^ were prepared, with pH adjusted to 7 and DO concentration maintained at 5 mg L^−1^. Antibiotic removal was performed using three different approaches: (1) immobilized *Chlorella* (IC) alone, (2) MNBs alone, and (3) a combination of both. The algal bead concentration was maintained at 1 bead per mL, while MNBs were introduced at a frequency of 10-min aeration every 48 h. The experiment lasted for 12 days, with antibiotic concentration and algal density measured every other day. All experiments were conducted under dark conditions and repeated in triplicate.

#### 2.4.2 Impact factor experiments

For the experiment of removing antibiotics from groundwater by IC, a certain amount of antibiotic solution was transferred to a 25 mL colorimetric tube, and a certain amount of algae strain was added in turn to adjust the pH and control the dissolved oxygen concentration of the system. Then, the concentration of micro–nano bubbles in the system was increased according to different requirements. Sampling was performed at different time points in the experiment, and the concentration of antibiotics was determined after filtration with a 0.22 μm filter membrane. The conditions of each influencing factor are set as follows:

The initial conditions were set with an antibiotic concentration of 10 mg L^−1^, algal bead concentration of 1 bead per mL, pH 7, DO 5 mg L^−1^, and aeration frequency of 10 min every 48 h. Antibiotic concentrations were measured before each aeration cycle. Unless otherwise specified, all experiments were conducted under these standardized conditions and repeated in triplicate.

Effect of initial antibiotic concentration: tested concentrations: 5, 10, 15, 20, and 30 mg L^−1^ of SD and CAP.

Effect of algal bead concentration: tested concentrations: 0.25, 0.5, 1, 2, and 4 beads per mL.

Effect of aeration duration: tested aeration durations: 5, 10, 15, 20, and 30 min every 48 h.

Effect of coexisting ions: prepared 2 L of simulated groundwater, with deionized water as a blank control. Added ion solutions (K^+^, Na^+^, Mg^2+^, Ca^2+^, HCO_3_^−^, NO_3_^−^, and SO_4_^2−^) at concentrations of 0, 2, and 5 mM.

#### 2.4.3 Experiments on antibiotic removal mechanisms

On day 12, 5 mL (approximately 96 beads) of immobilized algal beads were dissolved and centrifuged (5000 rpm, 10 min) to analyze antibiotic adsorption by the alginate carrier. The pellet was washed with 5 mL ultrapure water and recentrifuged to determine biological adsorption. For intracellular accumulation analysis, the cells were extracted with 5 mL dichloromethane-methanol (1 : 2 v/v) *via* ultrasonication (40 kHz, 2.2 kW, 1 h) followed by centrifugation.^36^

### 2.5 Measurement methods

#### 2.5.1 Methods for determining antibiotic concentrations

Filtered 2 mL samples through 0.22 μm membranes were analyzed using UPLC (Waters, USA) with an ACQUITY UPLC BEH C18 column (150 × 2.1 mm, 1.7 μm) at 0.2 mL min^−1^ flow rate and 2 μL injection volume. The mobile phase was methanol (LCMS grade) and aqueous solution containing 0.1% formic acid (10 : 90, v/v). The column temperature was 30 °C. The detection wavelengths of SD and CAP were 260 nm and 278 nm, respectively. LOQs were 0.03 mg per L (SD) and 0.05 mg per L (CAP), while LODs were 0.01 mg per L (SD) and 0.02 mg per L (CAP).

The removal process of antibiotics were analyzed by using pseudo-first-order kinetic model, which can be expressed as:^37^1
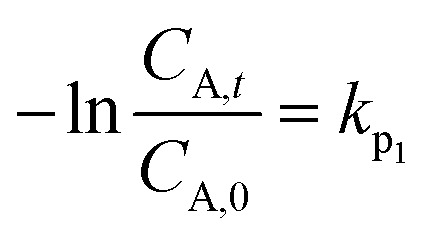
where *C*_A,*t*_ is the antibiotic concentration at time *t* (mg L^−1^), *C*_A,0_ is the initial concentration (mg L^−1^), and *k*_p_1__ is the pseudo-first-order rate constant.

The half-life equation:2
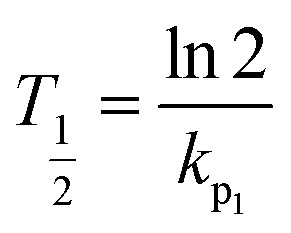


#### 2.5.2 Determination of algal cell biomass

Centrifuged algal cells were resuspended in sterile groundwater at different concentrations to determine algal biomass. Cell density was measured using an algal analyzer (Algapro 21E, Rike Environmental Technology Co., Wuhan) and confirmed by OD680 measurements (UV-2450, Shimadzu). Establish the standard curve of OD value and cell density *y* (cells per L) = 3.4185 × 10^9^*x* − 4.1848 × 10^8^ (*R*^2^ = 0.9786), and calculate the amounts of algae cells according to the standard curve. The inhibition rate of algal cell density was calculated as follows:^38^3
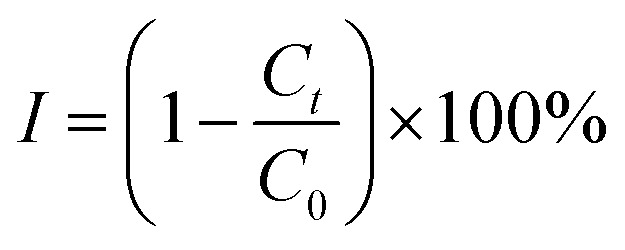
where *C*_*t*_ and *C*_0_ represent treatment and control group cell densities (cells per L), respectively.

#### 2.5.3 Determination of algal protein and polysaccharide content

Approximately 96 algal beads in a 5 mL suspension were centrifuged at 8000 rpm for 5 min to collect the biomass. The pellet was resuspended in 0.85% NaCl solution to the original volume, incubated at 60 °C for 30 min, then centrifuged (12 000 rpm, 15 min). The supernatant was filtered (0.22 μm) to obtain a crude EPS solution. Protein content was determined by the Coomassie Brilliant Blue method, while polysaccharide content was measured using the anthrone–sulfuric acid assay.^39^

#### 2.5.4 Correlation and principal component analysis (PCA)

Correlation analysis between influencing factors and antibiotic removal efficiency was performed using Origin 2024's Correlation Plot module and SPSS software. PCA was subsequently conducted to identify dominant influencing factors.

#### 2.5.5 SEM tests

Immobilized algal beads were fixed in 2.5% glutaraldehyde for 20 min, followed by gradient dehydration with 30%, 50%, 70%, 90%, and 100% ethanol (20 min per step, repeated twice). After overnight freezing at −80 °C, samples were lyophilized (YTLG-10A, Yetuo Technology, China) for 12 h. Gold-sputtered samples were examined by scanning electron microscopy (S-4800, Hitachi, Japan) for surface morphology.^40^

#### 2.5.6 FT-IR testing

Blank and antibiotic-treated immobilized algal beads were dried (60 °C, 24 h), ground (100-mesh sieve), and pelletized with KBr. Spectra (400–4000 cm^−1^) were acquired using an FTIR spectrometer (FTIR-650S, Guangdong Technology, China) in attenuated total reflection mode.^41^

## 3 Results and discussion

### 3.1 Algae performance

**Fig. 1:**
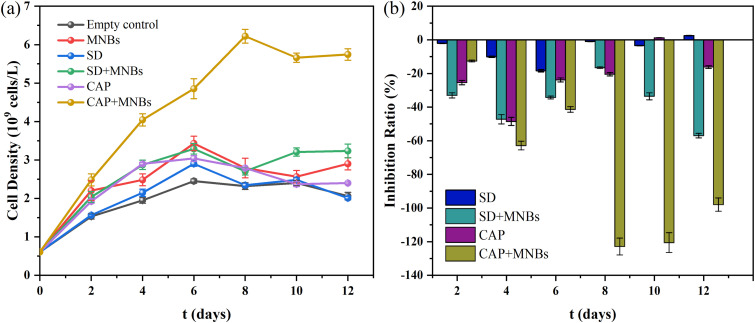
Growth curves of *C. vulgaris* under different conditions (a), and growth inhibition of antibiotic on *C. vulgaris* (b).

As shown in Fig. 1(a), *C. vulgaris* growth stabilized and reached its maximum on Day 6. When SD and CAP were added, the growth remained stable on Day 6. However, in the CAP + MNBs group, the maximum was reached on Day 8, possibly due to the synergistic effect of MNBs, which prolonged the growth cycle. This delay suggests that MNBs alleviated the toxic inhibition caused by CAP. At the end of the 12-day culture period, the number of algae in the SD + MNBs group was 3.235 × 10^9^, and the CAP + MNBs group was 5.745 × 10^9^. Compared to the SD group and the CAP group, the increases were 1.61 times and 2.4 times, respectively. MNBs significantly promoted the growth of *C. vulgaris* because MNBs not only increased the content of DO in water (Fig. SI4†), provided oxygen for the development of *C. vulgaris*, but also accelerated the transfer of nutrients in the water medium to organisms, thereby indirectly stimulating enzyme activity^42^ and promoting the growth and development of microorganisms.^43^

As can be seen from Fig. 1(b), the growth inhibition of *C. vulgaris* was negative in the first 8 days of the exposure period, and both antibiotics promoted algae growth to varying degrees. The CAP group demonstrated a promotional effect after a temporary inhibition observed on the 10th day, attributed to the degradation of antibiotics and the acclimatization of *C. vulgaris*. The SD group showed the weakest promotion effect and finally showed an inhibition effect. Because microalgae respond differently to different antibiotics,^36^ SD induces toxicity and exceeds the tolerance limit of the algal cells, destroying and disintegrating the cellular structure. In contrast, MNBs resulted in much higher algae populations when antibiotic concentrations were relatively low and acted as an exogenous carbon source to promote algae growth. Ma *et al.* (2024)^38^ showed that higher initial algal biomass alleviated the growth inhibition of *Chlorella* sp. by SDZ-induced stress.

### 3.2 Removal efficiency and kinetics

**Fig. 2:**
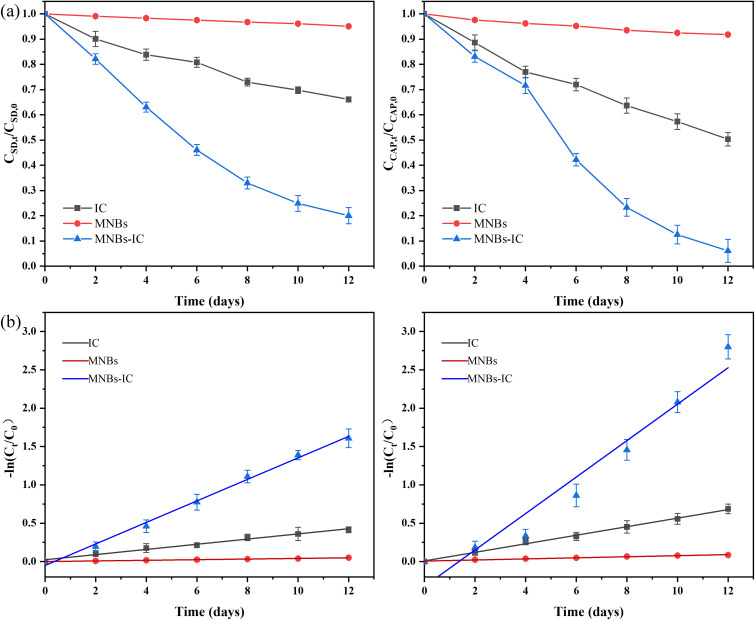
SD (left) and CAP (right) are removed over time in different systems (a) and kinetic fitting (b).

Fig. 2 illustrates the removal process and kinetic fitting of the SD and CAP in MNBs, IC, and MNBs-IC. The enhanced system shows the highest antibiotic removal rates, achieving 79.97% and 93.92%, respectively. Compared with the single IC system, the removal rate was significantly improved, increasing to 2.36 and 1.89 times, respectively. The collapsed MNBs can form a high-speed jet with high energy density, and the surrounding water molecules are pyrolyzed to produce hydroxyl radicals to degrade organic matter.^44^ However, Fig. 2 shows that the degradation effect of MNBs is significantly smaller than that of *C. vulgaris*. Combined with 3.1, we can conclude that MNBs primarily contribute to the growth of *C. vulgaris*.

The Table 2 below displays the kinetic parameters related to the antibiotic removal process. The pseudo-first-order model is typically applicable to diffusion-dominated rapid adsorption processes.^45^ The high water solubility of SD facilitates its adsorption onto the microalgae cell surface *via* hydrophobic interactions, resulting in liquid film diffusion acting as the rate-limiting step, which aligns well with the assumptions of the pseudo-first-order model.^46^ In contrast, the strong hydrophobicity of CAP leads to its removal relying on multi-step mechanisms, such as surface adsorption, chemical bonding, and biodegradation. These complex processes cannot be adequately described by the pseudo-first-order model. The removal efficiency of CAP is significantly higher than that of SD due to the strong specificity of algae in removing different antibiotics.^36^ Research indicates that the antibiotic removal rate is highly relevant to the alcohol/water partition coefficient (log *K*_ow_).^47^ The log *K*_ow_ values of CAPs and SD are 1.14 and −0.09, respectively. The larger the log *K*_ow_ value is, the higher the concentration of organic compounds in octanol is, indicating that the material is susceptible to passing through the phospholipid bilayer structure of the cell membrane and the easier it is to enter the cell. Moreover, the adsorption of antibiotics onto microalgae is affected by electrostatic attraction, which links to the ionization properties of the antibiotics.^48^ In this experiment, the pH value was maintained at around 7, making CAP primarily positively charged and SD predominantly negatively charged. Due to the presence of carboxyl, hydroxyl and amino groups on the cell membrane, the surface of *Chlorella* is usually considered to be negatively charged. MNBs are also negatively charged due to the presence of hydroxyl radicals at the interface and the electric double layer effect.^49,50^ Therefore, the electrostatic attraction between MNBs-IC system and CAP is stronger than that of SD, which further explains the higher removal rate of CAP.

**Table 2:** Kinetic parameters for antibiotics removal

	SD				CAP			
	*K* (d^−1^)	*t*_1/2_ (d)	*R* ^2^	Removal rate	*K* (d^−1^)	*t*_1/2_ (d)	*R* ^2^	Removal rate
IC	0.033	20.482	0.982	33.87%	0.054	12.914	0.991	49.67%
MNBs	0.004	168.066	0.997	4.90%	0.007	103.315	0.975	8.19%
MNBs-IC	0.140	4.972	0.995	79.97%	0.238	3.479	0.939	93.92%

### 3.3 Analysis of influencing factors and correlations

#### 3.3.1 Initial antibiotic concentration

**Fig. 3:**
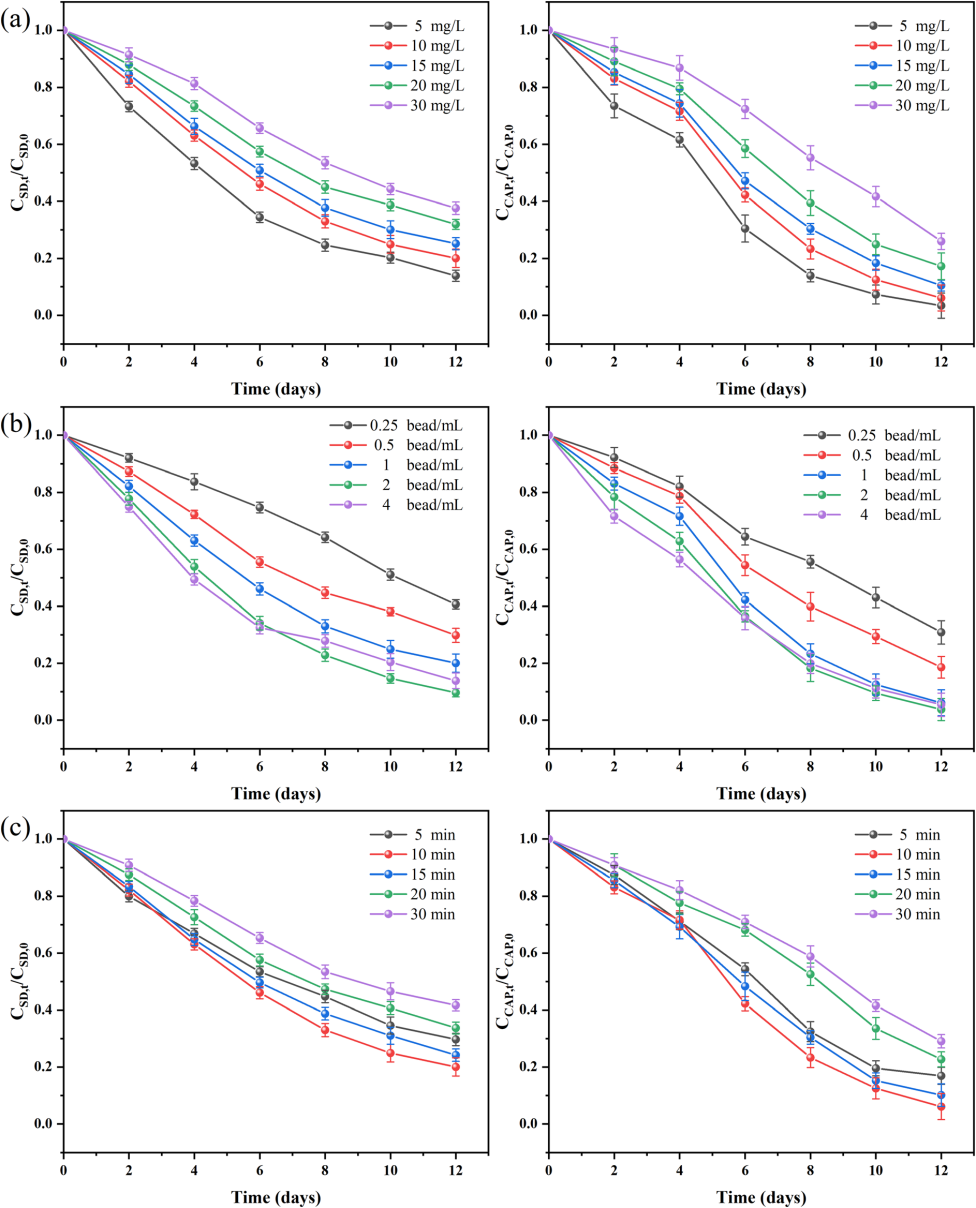
The effects of various influencing factors on the removal rate of SD (left) and CAP (right): (a) initial antibiotic concentration (b) algae concentration (c) effect of aeration time.

Analysis of Fig. 3(a) showed that both SD and CAP removal rates decreased with the increase of the initial antibiotic concentration. At the end of the 12-day experimental cycle, the removal rates of SD were 86.11%, 79.97%, 74.83%, 68.04%, and 62.35% at initial concentrations of 5, 10, 15, 20, and 30 mg L^−1^, respectively. Meanwhile, the removal rates for CAP reached 96.62%, 93.92%, 89.57%, 82.76%, and 74.02% at the same initial concentrations. The limited number of adsorption sites on the immobilized carriers means that sodium alginate and *C. vulgaris* are inadequate for higher concentrations of antibiotics. Higher concentrations of organic pollutants may also be toxic to microalgae, not only disturbing the homeostasis of reactive oxygen species to damage the cell structure and organelles^51^ but also decreasing the activity of adenosine triphosphate (ATP) synthase, which interferes with the energy conversion in the *C. vulgaris* mitochondria and chloroplasts, resulting in the inhibition of the growth and metabolism of the *C. vulgaris*.^52^ Meanwhile, excessive organic loading led to increased oxygen consumption, affecting the stability of MNBs and oxygen transfer efficiency.

#### 3.3.2 Algae concentration

Fig. 3(b) reveals that when the concentration of algal beads increased from 0.25 bead per mL to 2 bead per mL, the removal rate of antibiotics increased with the increase of algal beads concentration. However, when the concentration of algal beads further increased to 4 beads per mL, the removal rate of antibiotics decreased. The excessive number of IC leads to excessive competition of adsorption sites, which reduces the adsorption efficiency of each algal cell, thus affecting the overall degradation effect. Excessive algal growth may not be sustainable due to substrate concentration or oxygen inhibition. The CAP removal rate changed by 2.36% at 1 bead per mL, 2 bead per mL and 4 bead per mL, while the SD removal rate changed by 10.39%, indicating that the SD removal rate was sensitive to the concentration of algae beads.

#### 3.3.3 Aeration time

DO concentration was regulated by aeration time (Fig. SI4†). Achieved saturation DO (14.12 mg L^−1^) after 10 min of aeration, which corresponded with peak antibiotic removal efficiency (Fig. 3(c)). Within the studied range, removal rates of both antibiotics showed positive correlations with DO levels. Within the scope of the study, the removal rate of both antibiotics was positively correlated with oxygen concentration, which was the result of the synergistic effect of oxygen supply, mass transfer efficiency, biological metabolism and chemical oxidation. MNBs increase DO concentration through efficient mass transfer, directly promote the microbial activity of *Chlorella*, and accelerate the biomass accumulation of *Chlorella*.^53^ At the same time, the increase in oxygen concentration increased ·OH production efficiency promotes antibiotic degradation. To maintain experimental consistency and prevent thermal stress on *C. vulgaris*, all aerated solutions were cooled to room temperature before being reintroduced to the sample bottles containing immobilized algal beads.^54^

#### 3.3.4 Coexisting ions

**Fig. 4:**
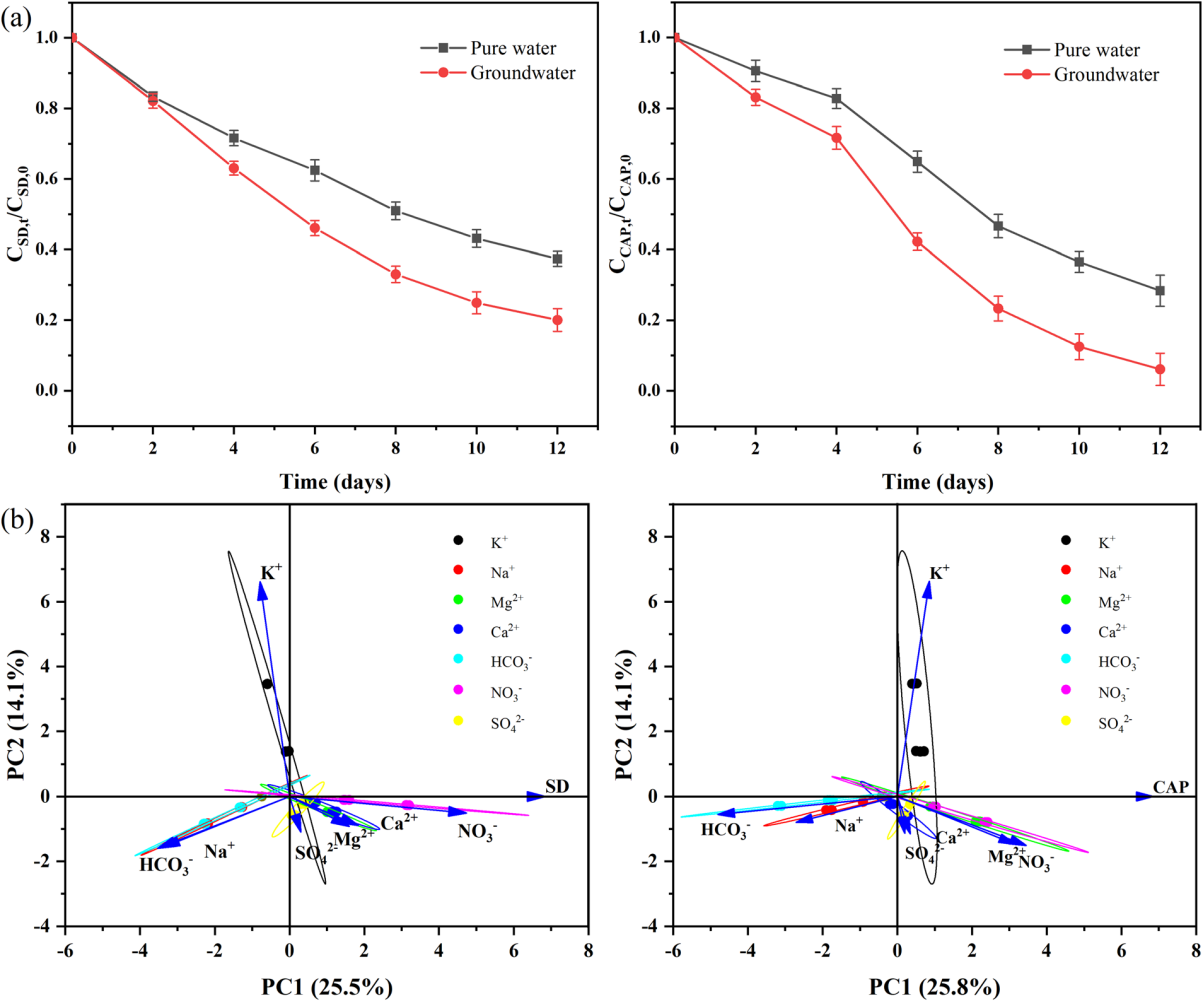
Effect of coexisting ions on the removal of antibiotics SD (left) and CAP (right) (a) and PCA (b).

It is well known that natural groundwater contains many ions, such as K^+^, Na^+^, Mg^2+^, Ca^2+^, HCO_3_^−^, NO_3_^−^ and SO_4_^2−^. Fig. 4(a) showed coexisting ions significantly sweetened the removal of antibiotics. Most of these ions serve as nutrients for algae growth,^55,56^ and high concentrations of inorganic ions are prone to salting out, increasing the concentration of organic matter gathered on the surface of the bubbles^57^ and forming an environment conducive to the degradation of antibiotics. The removal rate of antibiotics across different ions and the concentrations of three ions were analyzed using PCA, as shown in Fig. 4(b), to evaluate the role of individual ions. NO_3_^−^ was the most substantial ion contributing to the first principal component (PC1), positively correlated with the SD removal rate. HCO_3_^−^ and Na^+^ also significantly contributed to PC1 and were negatively correlated with the removal rate. The primary contributor to the second principal component (PC2) was K^+^, which showed a significant positive correlation with the removal rate. Unlike SD, HCO_3_^−^ was the ion that contributed most significantly to PC1 and exhibited a significant negative correlation with the CAP removal rate. As the nitrogen source of microalgae, nitrate can be converted into ammonia nitrogen by nitrate reductase and nitrite reductase, which can be assimilated and utilized by microalgae. HCO_3_^−^ can selectively dissipate the proton dynamic force along the pH gradient of the cell membrane, reduce the absorption and retention of antibiotics,^58^ and reduce the hydroxyl radicals in the solution,^59^ which is not conducive to the removal of antibiotics.

#### 3.3.5 Correlation analysis

**Fig. 5:**
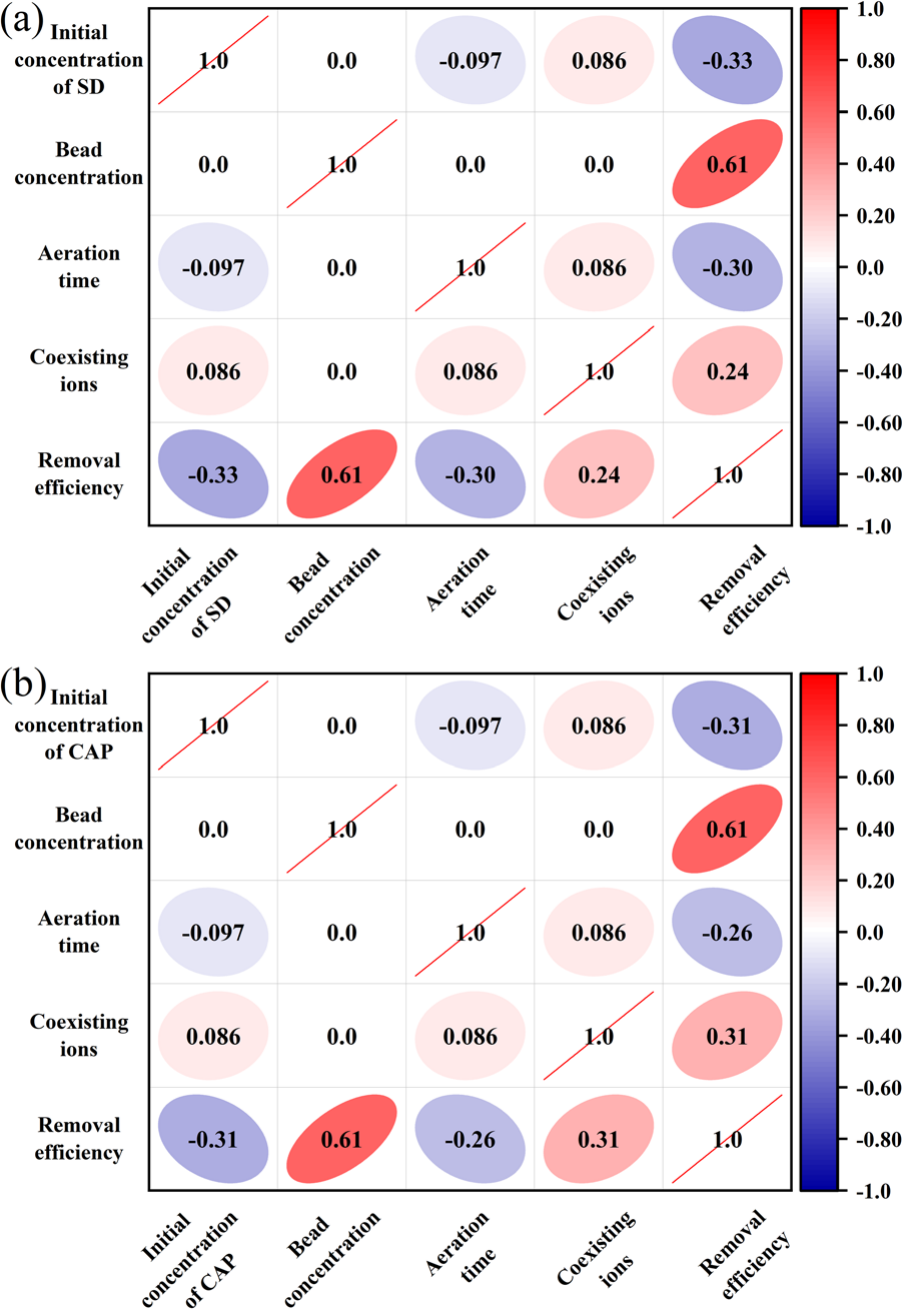
Correlation analysis (a) and PCA plot (b) between antibiotics removal rate and factors.

Determine the dominant factors affecting antibiotic removal rate by correlation analysis to clarify the relationship between various influencing factors and antibiotic removal rate. Correlation analysis used the Spearman correlation coefficient. According to Fig. 5, the removal rates of both antibiotics showed a negative correlation with aeration time and the initial antibiotic concentration, a strong positive correlation with the concentration of algal beads (*r* = 0.61), and a positive correlation with coexisting ions. Among them, the initial concentration of antibiotics and aeration time have a powerful effect on SD, and the influence of coexisting ions on CAP is keener.

The effectiveness of antibiotic removal based on microalgae varies with the type of antibiotics and microalgae species.^60^ Microalgae are more effective in removing macrolide antibiotics than β-lactam and sulfonamide antibiotics, with an overall removal rate of 62.3%.^61^ Chen *et al.* (2020)^62^ studied that the removal rate of 10 mg per L SD by *C. vulgaris* was only 29%. Wang *et al.* (2023)^46^ used *Chlorella* pyrenoidosa to remove SD at different initial concentrations (100, 200 and 500 μg L^−1^). The removal efficiency is 65.9–67.6%. The method used in this study has obvious advantages in removal efficiency.

### 3.4 Antibiotic removal mechanisms

SEM analysis revealed distinct morphological differences in immobilized *C. vulgaris* after 12-day cultivation (Fig. SI5†). Control group cells exhibited extensive rupture and mortality, with wrinkled and distorted surfaces. In contrast, antibiotic-exposed cells maintained smoother surfaces and intact morphology, particularly in CAP-treated samples, which showed optimal cellular integrity and higher cell density. These observations suggest that nutrient limitation (carbon/nitrogen sources) in the control group induced algal senescence, while SD and CAP potentially served as supplemental nutrients promoting algal growth. These findings are consistent with the growth trends observed in Section 3.1.

#### 3.4.1 Infrared spectral characterization

**Fig. 6:**
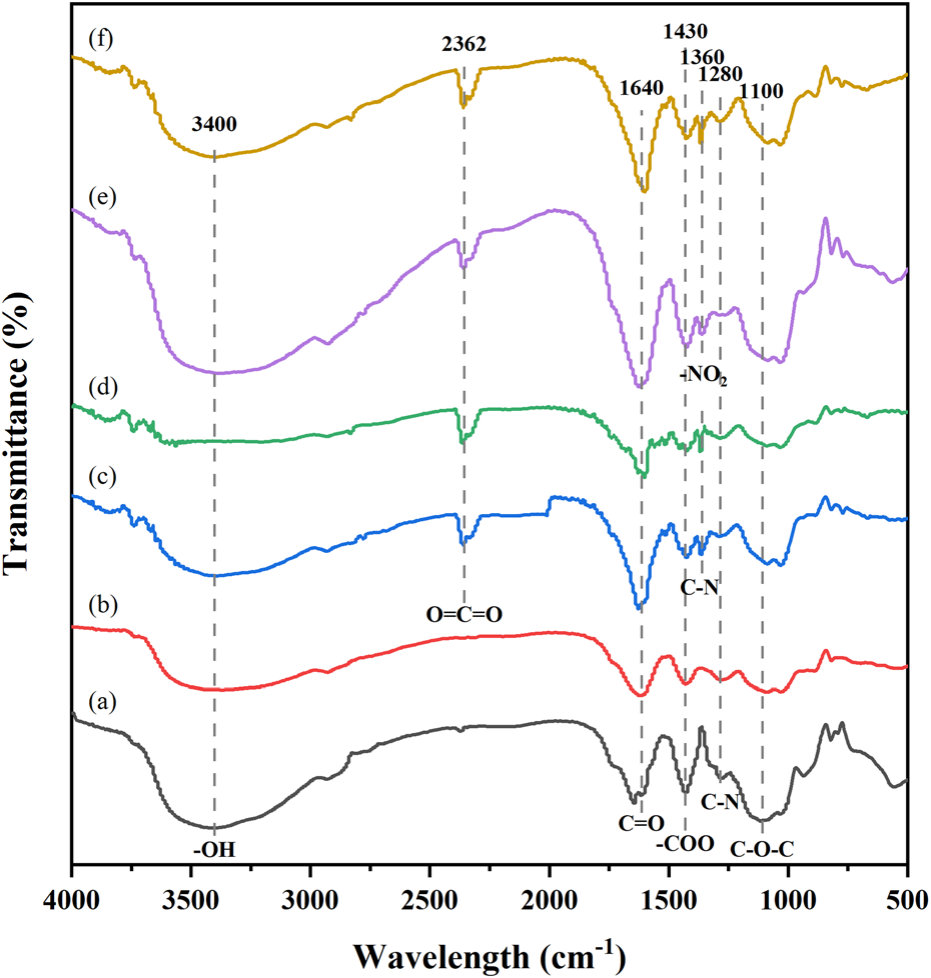
FT-IR images of immobilized *C. vulgaris* beads treated under different conditions for 12 days: (a) hypoxia-no antibiotics; (b) MNBs-no antibiotics; (c) hypoxia-SD; (d) MNBs-SD; (e) hypoxia-CAP; (f) MNBs-CAP.

FT-IR spectral characterization revealed that MNBs did not induce new functional groups but caused characteristic peak shifts (10–40 cm^−1^) in immobilized *C. vulgaris*, suggesting van der Waals or hydrogen bonding interactions between antibiotics and the algal matrix.^63^ As shown in Fig. 6, CAP demonstrated more significant metabolic/binding interactions with algal cells than SD. The absorption peaks in the 3600–3300 cm^−1^ range, attributed to –NH and –OH stretching vibrations,^64^ showed noticeable shifts in antibiotic-treated groups, indicating the potential involvement of these groups in adsorption or removal processes. A new peak emerged near 2362 cm^−1^, likely corresponding to O=C=O stretching vibrations. The peak at 1430 cm^−1^, assigned to –COO– symmetric stretching,^33^ exhibited minimal shift, suggesting limited participation in antibiotic removal. Significant peak shifts were observed at 1640 cm^−1^ (amide I–C=O stretching) and 1100 cm^−1^ (–C–O–C stretching).^65^ A new peak appeared at 1360 cm^−1^ in all antibiotic groups, which was attributed to the symmetric stretching vibration of –NO_2_ in CAP group, ^66^ and may reflect the C–N stretching of the degradation product 2-aminopyrimidine in SD group.^67^ The peak at 1271.8 cm^−1^ may be the stretching vibration peak of the C–N of the amide III band. These findings suggest that functional groups such as –NH, –OH, –C=O, C–N, and –C–O–C in *C. vulgaris* likely participate in the enhanced removal of antibiotics by MNBs, but the specific mechanisms require further investigation.

#### 3.4.2 Antibiotic removal pathways

**Fig. 7:**
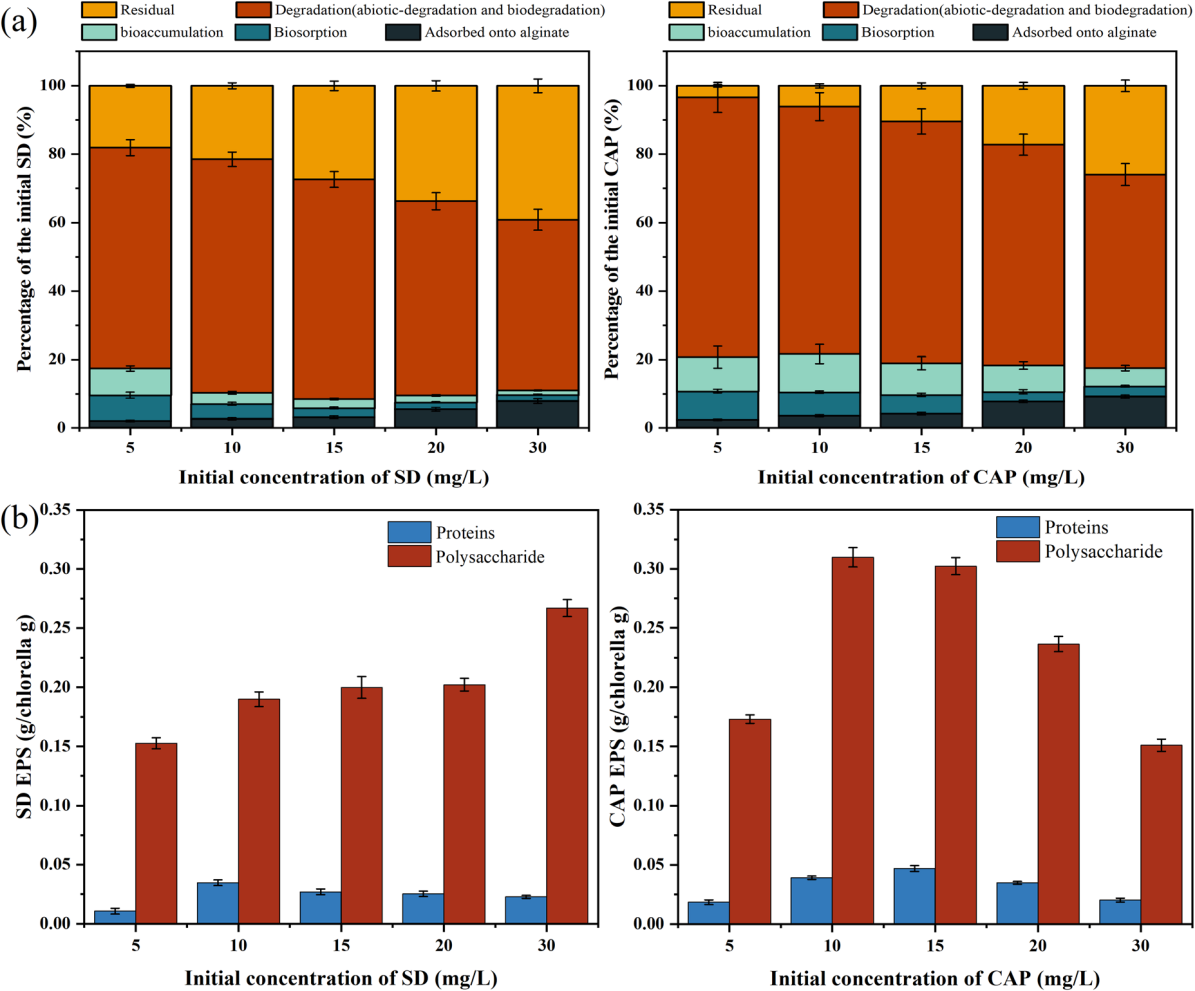
Mechanism of antibiotic removal: (a) percentage of removal pathways for different concentrations of antibiotics (b) EPS content of *C. vulgaris*.

**Fig. 8:**
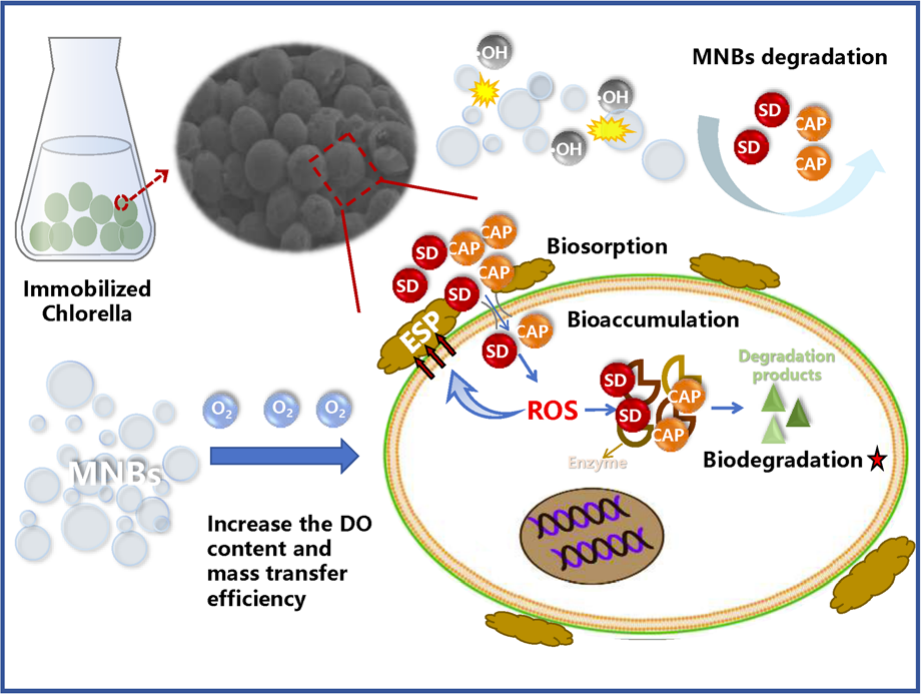
Mechanism diagram of antibiotic removal by IC enhanced by MNBs.

In the MNBs-IC system, antibiotics first migrated from the aqueous phase to the solid phase into the immobilized microalgae. The pathway of antibiotic removal in the whole process involves the adsorption of immobilized carriers, degradation of *C. vulgaris*, degradation of MNBs, and hydrolysis of antibiotics itself. The role of hydrolysis in the antibiotic removal process is small and negligible. The main removal pathway was degradation based on Fig. 7(a), with 49.85% to 68.20% for SD and 56.49% to 75.90% for CAP. Bioaccumulation, biosorption, and adsorption by immobilized materials contributed less. The findings of multiple studies that established biodegradation as the primary mechanism of action for microalgae's removal of antibiotics were in line with this.^21,51^ The fact that CAP had a greater capacity for biosorption than SD is also evident. Studies have shown that surface adsorption of *Chlorella* is a key factor in determining the biodegradation efficiency of antibiotics,^68^ and different biosorption amounts may have led to different biodegradation efficacy of antibiotics. The mechanism of MNBs enhancing the removal of antibiotics by IC is shown in Fig. 8. Biodegradation depends on various intracellular and extracellular enzyme active substances. MNBs produce hydroxyl radicals to play a certain role in degradation. At the same time, by increasing dissolved oxygen content and promoting mass transfer, the growth of algal cells is accelerated, thereby degrading antibiotics.

In addition to the nature of antibiotics, biosorption is also affected by hydrophobic mechanisms, such as EPS.^69^ ESP is mainly composed of proteins and polysaccharides. There are many forces in the process of binding with antibiotics, including the van der Waals force, hydrogen bond, and electrostatic attraction.^70^ With the increase in antibiotic concentration, the ESP of *C. vulgaris* in the SD group increased to varying degrees, while that in the CAP group increased first and then decreased. This may be caused by the combined effect of the biomass of *C. vulgaris* and the stress of antibiotics on *C. vulgaris*.^10^ Wang *et al.* (2019)^71^ studies have shown that high concentrations of antibiotics can stimulate *Chlorella* to secrete ESP to protect and maintain cell activity. We found that the content of EPS well correlated with the biosorption ratio of algae cells combined with the removal pathway, especially in the CAP group. Because of its strong hydrophobicity and chemical makeup, CAP facilitated chemical bonding and physical adsorption by algal cell walls and EPS.^72^ According to the literature report,^73^ EPS affects the adsorption of antibiotics by *Chlorella* mainly through the –C=O, –NH_2_, and –OH of proteins and the C–O–C functional groups of polysaccharides, which can provide more active sites to adsorb antibiotics, corroborated by FT-IR results in Section 3.4.1.

## 4 Conclusions

MNBs can promote *C. vulgaris* growth by increasing the amount of DO in water and significantly enhancing the removal efficiency of antibiotics by the IC. The system has a firmer enhancement effect on the SD removal process. The removal process followed the first-order kinetic model. The experimental results of various influencing factors showed that the toxic effect of SD on *C. vulgaris* may be greater than that of CAP; algae concentration is the most significant factor affecting the removal rate of antibiotics. Aeration time affects the removal effect by influencing the DO value in water, and the coexisting ions in the groundwater promote the removal of antibiotics. Initial antibiotic concentration and aeration time had a more pronounced effect on SD removal, and coexisting ions had a more potent impact on CAP. Both antibiotic removal pathways include adsorption, biosorption, bioaccumulation, and degradation (biodegradable and non-biodegradable) of immobilized carriers. Degradation was the primary mechanism among them, and the amount of adsorption was the primary determinant of antibiotic degradation efficiency. ESP and the antibiotics' nature worked together to give CAP a higher degradation efficacy than SD.

## Author contributions

Tao Zhu: writing – original draft, funding acquisition, formal analysis. Mengyao Jing: writing – original draft, supervision, methodology. Jianping Zhang: writing – review & editing, supervision, data curation. Hui Li: writing – review & editing, data curation. Min Zhou: writing – review & editing, data curation. Guijuan Li: writing – review & editing, supervision, methodology, conceptualization.

## Conflicts of interest

There are no conflicts to declare.

## Supplementary Material

RA-015-D5RA02082D-s001

## Data Availability

The data that support the findings of this study are available from the corresponding author upon reasonable request.
