# Supplementary material for: Efficacy of micro–nano bubble enhanced immobilized Chlorella vulgaris in the removal of typical antibiotics
Source: RSC Adv. 2025 Jun 16;15(25):20268–80. doi: 10.1039/d5ra02082d (PMC12168768; doi:10.1039/d5ra02082d)
Supplement: RA-015-D5RA02082D-s001 [file RA-015-D5RA02082D-s001.pdf]

Supplementary Information (SI) for RSC Advances.
This journal is © The Royal Society of Chemistry 2025

Supporting information

Efficacy of Micro-nano bubble enhanced immobilized Chlorella vulgaris in the removal of typical antibiotics

Tao Zhu[a], Mengyao Jing [b,c,d], Jianping Zhang [b,c,d], Hui Li [e], Min Zhou [f,g], Guijuan Li [b,c,d]

[a] Henan College of Transportation, Zhengzhou 450008, Henan, China

[b] School of Water and Environment, Chang' an University, Xi'an 710054, China

[c] Key Laboratory of Subsurface Hydrology and Ecology in Arid Areas, Ministry of Education, Chang' an University, Xi'an 710054, China

[d] Key Laboratory of Eco-hydrology and Water Security in Arid and Semi-arid Regions of Ministry of Water Resources, Chang' an University, Xi'an 710054, China

[e] Henan Transport Investment Group Co., Ltd., Zhengzhou, China

[f] Ocean University of China, Qingdao 266100, Shandong, China

[g] Henan Provincial Department of Transport, Zhengzhou 45000, Henan, China

270426826@qq.com (T.Z.); 135935924967@163.com (M.J.); jianping29@126.com (J.Z.); 289868741@qq.com (H.L); zhoumin2023@126.com (M.Z.); changandxyl@163.com (G.L.).

***Correspondence**

Email: zhoumin2023@126.com.

Contains

Number of texts in SI: 1

Number of figures in SI: 5

Number of tables in SI: 1

Texts:

Text SI1 introduces the basic characteristics of micro-nano bubbles.

Figures:

Fig. SI1: Micro-nano-bubbles generator

Fig. SI2 displays the particle size distribution of micro-nano bubbles.

Fig. SI3 shows the stability of micro-nano bubbles.

Fig. SI4 shows the oxygen-increasing ability of micro-nano bubbles.

Fig. SI5 displays scanning electron microscope characterization of *C. vulgaris.*

Tables:

Table SI1 introduces the BG-11 medium formula.

Text SI1 Basic characteristics of micro-nano bubbles

The particle sizes of MNBs with different aeration times were significantly different. The experiments' micro-nano-bubble generator produced MNBs with particle sizes mostly concentrated between 200 and 500 nm. A previous study found a good linear relationship between the turbidity of MNB water and the concentration of bubbles obtained by resonance mass meter measurement, and the turbidity can be used to represent the concentration of bubbles in MNB water [1]. Therefore, it can be known from Fig. SI4. that the concentration of MNBs increases with the increase of aeration time, and a certain amount of MNBs still exists in the MNBs water at all five aeration times after sealed storage for 120 h. This suggests that MNBs can endure for a considerable amount of time with a certain level of stability.

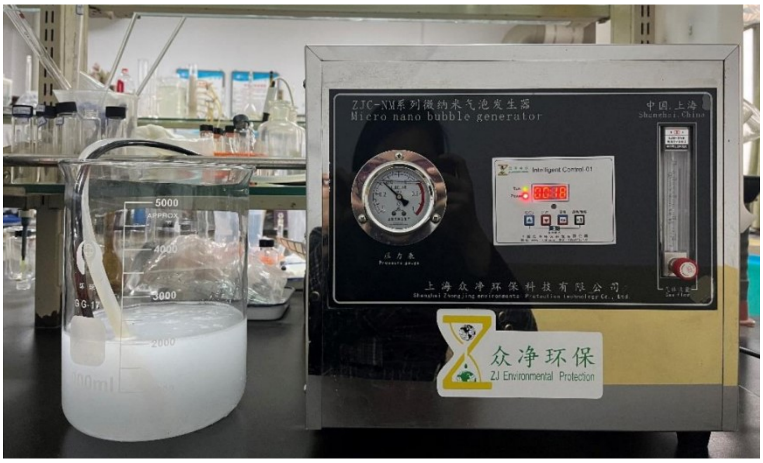

Fig. SI1: Micro-nano-bubbles generator

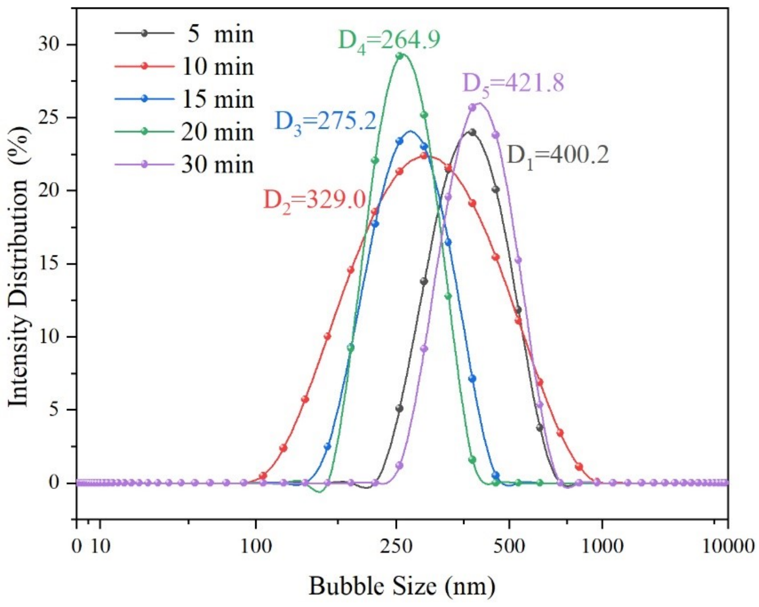

Fig. SI2: The size of MNBs

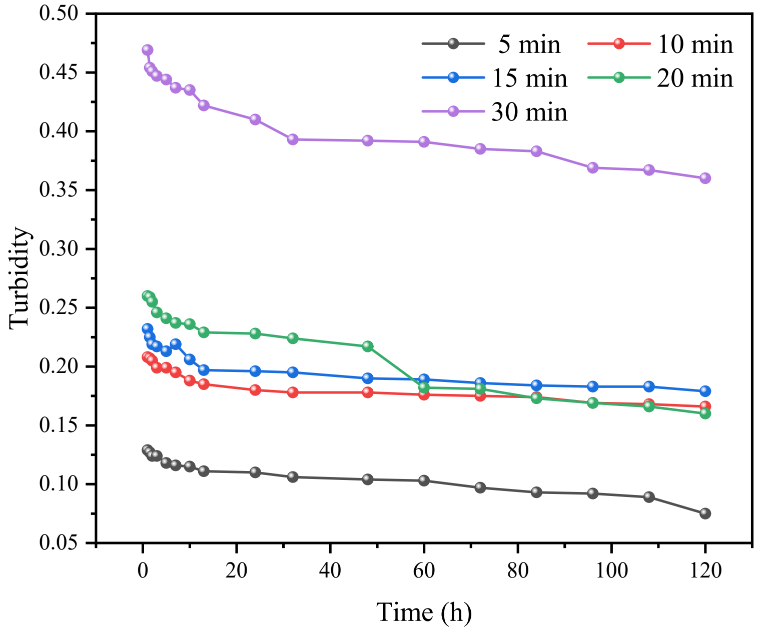

Fig. SI3: Changes in turbidity of MNBs water with time

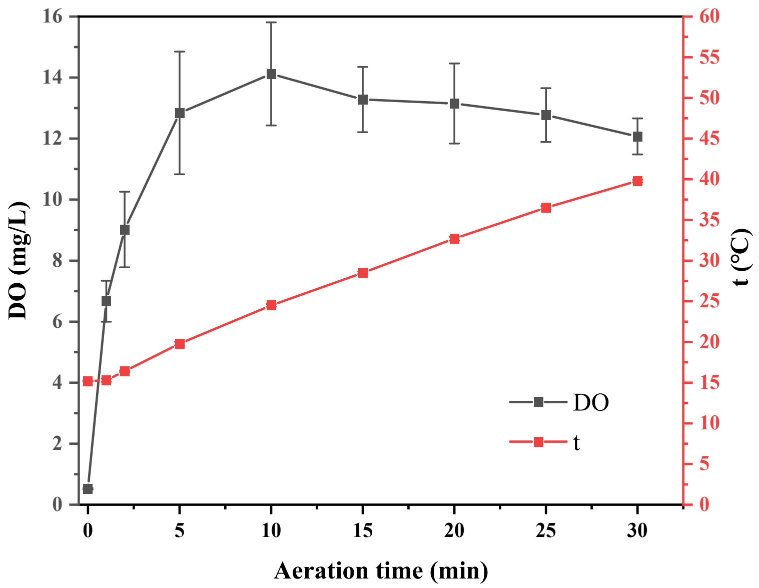

Fig. SI4: Trends in the oxygenation capacity of MNBs

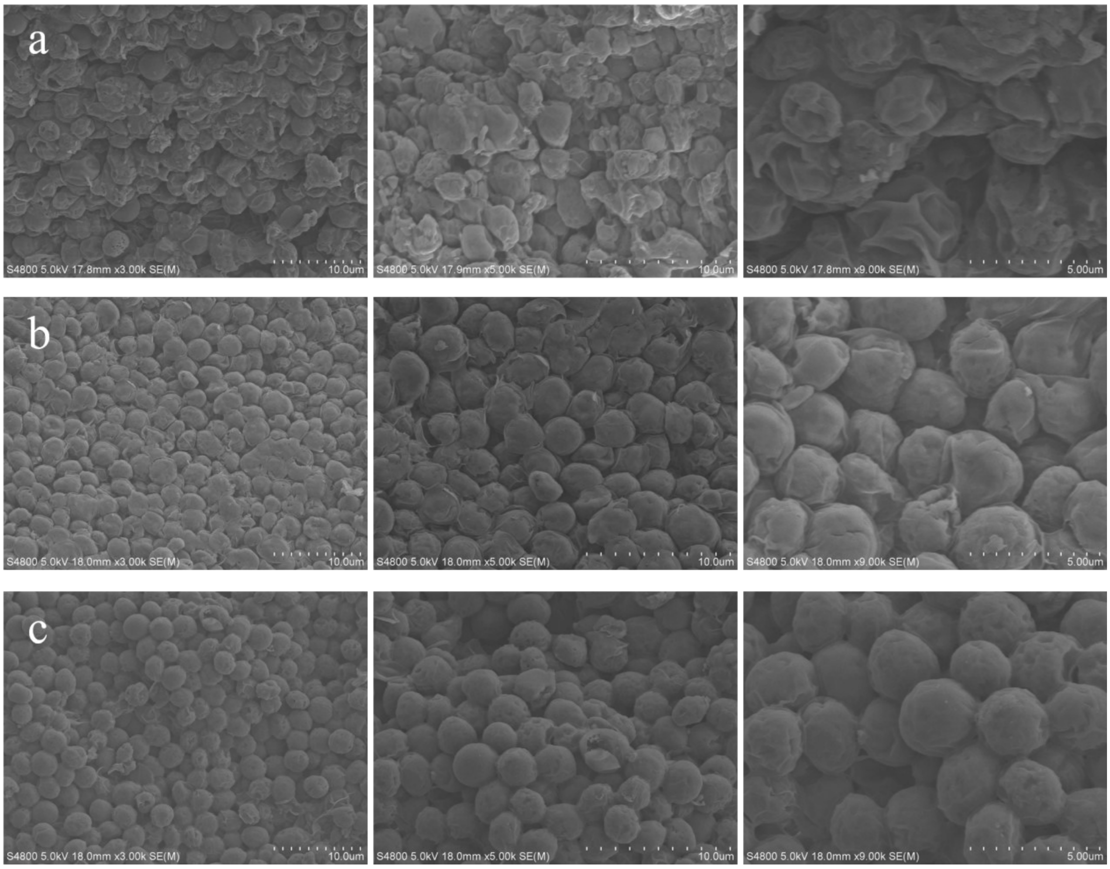

Fig. SI5: SEM images of immobilized Chlorella vulgaris growing in antibiotic-free(a), SD(b) and CAP(c) solutions for 12 days, respectively (magnification of each column from left to right is 3000×, 5000×, 9000×)

Table SI1 BG-11 medium formula

Chemical composition	Concentration(g/L)	Chemical composition	Concentration(g/L)
$NaNO_3$	1.5	Na_2CO_3	0.02
$K_2HPO_4 \cdot 3H_2O$	0.04	H_3BO_3	0.00286
$MgSO_4 \cdot 7H_2O$	0.075	$MnCl_2 \cdot H_2O$	0.00181
$CaCl_2 \cdot 2H_2O$	0.036	$ZnSO_4 \cdot 7H_2O$	0.000222
$C_6H_8O_7$	0.006	$CuSO_4 \cdot 5H_2O$	0.000079
$C_6H_8FeNO_7$	0.006	$Na_2MoO_4 \cdot 2H_2O$	0.00039
EDTA	0.001	$Co(NO_3)_2 \cdot 6H_2O$	0.000049

References

1 S. Hamamoto, T. Takemura, K. Suzuki, T. Nishimura, Effects of Ph on Nano-Bubble Stability and Transport in Saturated Porous Media, Journal of Contaminant Hydrology 208 (2018) 61-67, https://doi.org/10.1016/j.jconhyd.2017.12.001.
